# Remote Doping Effects of Indium–Gallium–Zinc Oxide Thin-Film Transistors by Silane-Based Self-Assembled Monolayers

**DOI:** 10.3390/mi12050481

**Published:** 2021-04-23

**Authors:** Juhyung Seo, Hocheon Yoo

**Affiliations:** Department of Electronic Engineering, Gachon University, Seongnam 13120, Korea; qaz4317@gachon.ac.kr

**Keywords:** SAM, IGZO transistors, N-doping, OTS, ODTS, carbon-chain, doping effect control

## Abstract

Oxide thin-film transistors (TFTs), including indium–gallium–zinc oxide (IGZO) TFTs, have been widely investigated because of their excellent properties, such as compatibility with flexible substrates, high carrier mobility, and easy-to-fabricate TFT processes. However, to increase the use of oxide semiconductors in electronic products, an effective doping method that can control the electrical characteristics of oxide TFTs is required. Here, we comprehensively investigate the effect of silane-based self-assembled monolayer (SAM) doping on IGZO TFTs. Instead of a complex doping process, the electrical performance can be enhanced by anchoring silane-based SAMs on the IGZO surface. Furthermore, differences in the doping effect based on the structure of SAMs were analyzed; the analysis offers a systematic guideline for effective electrical characteristic control in IGZO TFTs.

## 1. Introduction

Oxide semiconductors have been promising for various applications, e.g., thin-film transistors (TFTs) for flexible [1,2,3] and transparent [2] display products, photodetectors [3,4], and embedded sensors [5]. Compared with amorphous silicon (a-Si), oxide semiconductors enable easy-to-fabricate processes and incur lower fabrication costs, accompanied by a reduced thermal budget required for three-dimensional integrations. As an additional advantage, various morphological structures, such as nanoparticles [6], quantum dots [7,8], and nanowires [9,10], can be developed to allow oxide semiconductors to be used as high-aspect-ratio sensing layers [10]. Among oxide semiconductors, indium gallium zinc oxide (IGZO) is the most promising candidate for next-generation applications in various fields, such as active-matrix flat panel displays [11,12,13], new-concept switching transistors [14], and neuromorphic devices [15,16]. In particular, over the past decade, several research developments pertaining to IGZO-based devices have been reported owing to excellent carrier mobility [17] with the aforementioned advantages.

Despite the advantages of oxide semiconductors, e.g., IGZO, it has been a challenge to accurately control oxide-based TFT characteristics. Conventional silicon-based metal-oxide-semiconductor field-effect transistors (MOSFETs) can control device characteristics (i.e., threshold voltage (V_TH_) and carrier mobility (µ)) by doping, i.e., ion implantation. However, appropriate doping technologies compatible with oxide semiconductors have not been well-developed. The optimization and control of device characteristics were reported by controlling the thickness [18] of oxide semiconductors or deposition parameters (i.e., temperature [19], pressure [20], or gas rate [1,21,22,23]). In addition, as reported previously, inserting components such as Ga, Sn, and Si into oxide semiconductor bulks helped tune device parameters [24]. Remote doping was proposed as the third approach. By coating or depositing an additional layer, an increase or decrease in charge transport in the semiconductor can be tuned. Self-assembled monolayers (SAMs) have been used as remote doping layers [25]. They have molecular assemblies that form a chemical bond on the oxide surface, enabling a highly uniform and oriented domain morphology. In spite of the trial of SAM-based doping on oxide semiconductors, accurate control of doping effects and improvisation are still required.

Here, we present a systematic control of the SAM-based doping technique based on molecular chain length control and annealing temperature conditions (T_A_ = 120, 150, and 200 °C). We fabricated octyltrichlorosilane (OTS) and octadecyltrichlorosilane (ODTS)-treatment-doped IGZO TFTs. With an increase in T_A_, we investigated the electrical characteristics of the TFTs, including the carrier mobility, V_TH_, subthreshold swing (SS), and on–off current ratio. This method can help control electrical properties. The length of the carbon chain was modulated. Furthermore, we investigated the mechanism of the OTS and OTDS doping effects by means of surface energy extraction and contact resistance analysis.

## 2. Materials and Methods

### 2.1. Device Fabrication

**Figure 1:**
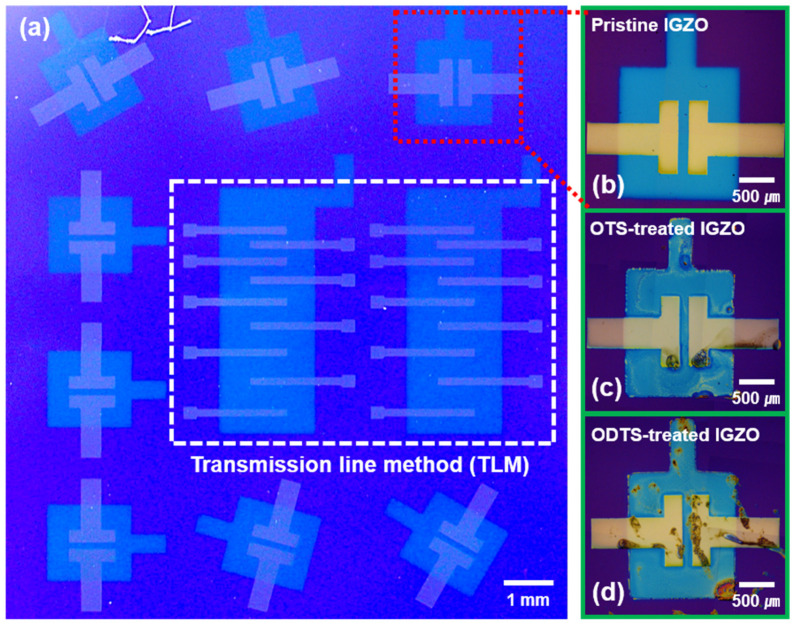
Image of transistor: (**a**) Optical microscopy image of IGZO TFT designed for experiment; (**b**) image of pristine TFT; (**c**) image of IGZO TFT treated with OTS; (**d**) TFT image with ODTS treatment.

The device exhibited an IGZO semiconductor-based top-contact bottom-gate structure. Figure 1a shows an optical microscope (OM) image of the devices. The SiO_2_/Si surface was cleaned and sonicated in 99% acetone for 20 min. IGZO was deposited by sputtering on a SiO_2_/Si substrate at 9.33 mbar in pure argon using 60 W of plasma. Then, 50 nm of titanium was deposited as a source/drain using e-beam evaporation. All the evaporation materials were patterned using a metal shadow mask. The channel length (L) and width (W) of the IGZO TFT were 100 μm and 1000 μm, respectively.

### 2.2. SAM Treatments

**Figure 2:**
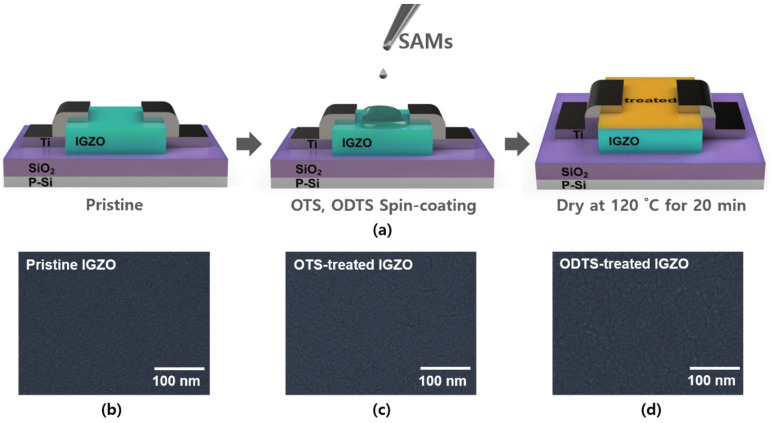
(**a**) Schematic of SAM treatment processes of IGZO-based transistor; (**b**–**d**) SEM image of the IGZO surface with the SAM treatment after dry at 120 °C.

OTS (Sigma-Aldrich St. Louis, MO, USA) and ODTS (Sigma-Aldrich) have additional 8 and 18 carbon chains in trichlorosilane (HCI_3_Si), respectively. We prepared 5 mL of a 1% *v*/*v* solution by mixing OTS and chlorobenzene (C_6_H_5_Cl) (Sigma-Aldrich) [26]. Moreover, ODTS was mixed using the same method with a 1% *v*/*v* concentration. The solutions were mixed for 6 h at room temperature using a magnetic stirrer. To perform the SAM treatment on top of IGZO surfaces, we applied UV-ozone treatment to the IGZO surface. UV-ozone treatment induces covalent bonding between the IGZO surface and trichlorosilane by forming OH on the IGZO surface. A solution of OTS and ODTS was spin-coated at 3000 rpm for 10 s on the IGZO surface where OH was formed. After the coating was complete, the TFT was dried in air for 20 min on a hot plate at 120 °C [25]. These processes were simply shown in Figure 2a.

### 2.3. Annealing Process and Characterization

The samples were annealed in air for 30 min at T_A_ = 150 and 200 °C and given enough time to cool down to room temperature before measurement. All measurements were performed in air at room temperature with a Keithley 4200 semiconductor parameter analyzer.

## 3. Results and Discussion

### 3.1. Electrical Properties of IGZO-Based TFTs by SAM Doping Effects

First, we prepared an IGZO-based TFT for SAM treatment. The semiconductor IGZO was deposited on Si/SiO_2_ via sputtering. Then, the source and drain were deposited using the e-beam evaporation method. Before spin-coating, we performed the UV-ozone treatment to attach SAM to the IGZO surface. The OTS and ODTS solutions were spin-coated on the IGZO surface (Figure 2a). Figure 1b–d shows the OM images of the pristine IGZO, OTS-treated IGZO, and ODTS-treated IGZO TFT, respectively. We also characterized the surfaces of the respective devices using a scanning electron microscope (SEM) to investigate the morphological change by the SAM treatment. Figure 2b–d shows the SEM images of the pristine IGZO, OTS-treated IGZO, and ODTS-treated IGZO, respectively, which exhibited negligible variation due to the thin nature of the SAM layer [27].

**Figure 3:**
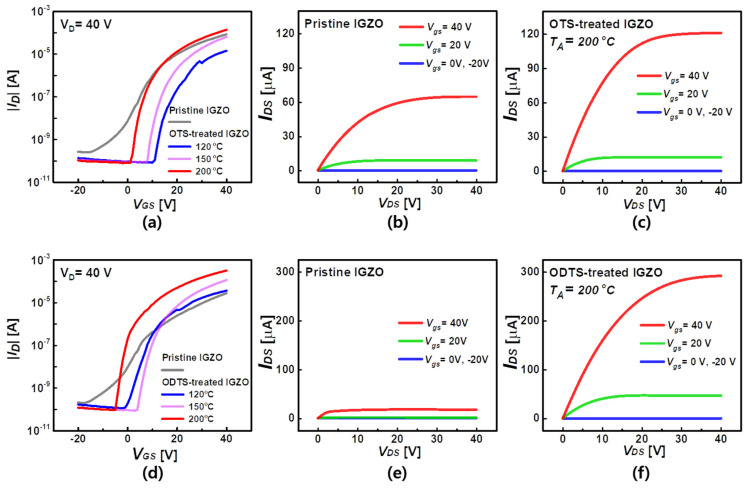
Transfer curve based on the annealing temperature of OTS–treated (**a**) and ODTS–treated transistors (**d**); Pristine output curve of OTS–treated (**b**) and ODTS–treated transistors (**e**); Output curve of OTS–treated (**c**) and ODTS–treated transistors (**f**) according to annealing (T_A_ = 200 °C).

**Figure 4:**
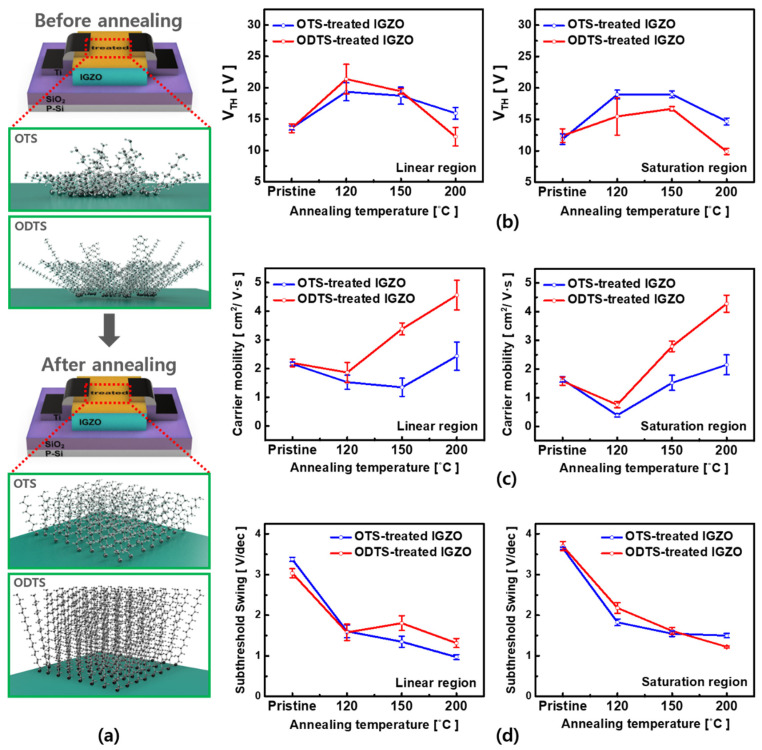
(**a**) Structural changes in the carbon chain according to heat treatment (annealing); structures are aligned after heat treatment. Variation of V_TH_ (**b**), effective carrier mobility (**c**), SS (**d**) according to annealing and treatment.

Next, we investigated electrical characteristics of the fabricated TFTs depending on the SAM’s carbon change length and T_A_. We measured both transfer and output characteristics of the devices, and, after the respective SAM treatment (i.e., OTS or ODTS), we measured the corresponding devices under the same measurement condition (Figure 3a–f). Compared to the measured electrical characteristics of the pristine IGZO TFT, the OTS-treated IGZO TFT at T_A_ = 120, 150 °C provided the increase of the V_TH_ and accordingly, the decrease of the on-current (at V_G_ = 40 V). However, at T_A_ = 200 °C, we observed an enhancement of the electrical characteristics; the on-current was doubled (64–121 µA) (Figure 3b,c). The ODTS-treated IGZO at T_A_ = 200 °C showed further improved characteristics. To be specific, the on-current was improved (×5.38 times higher from 40 ± 9.27 µA to 214 ± 0.37 µA) compared to the counterpart of the pristine (Figure 3d–f). This effect was caused by the alignment of trichlorosilane carbon chains (Figure 4a). When the carbon chains are aligned by the high temperature annealing, a dipole is formed on the IGZO surface, and electrons are injected into the channel, increasing the on-current.

To verify reliability and uniformity of the SAM treatment doping effects, we performed the statistical experiments by measuring 8 devices for each condition. Figure 4b–d show the trend of the device parameters (i.e., V_TH_, effective carrier mobility, and SS) as a function of T_A_ and the SAM carbon chain length. Interestingly, after the SAM treatment, the OTS and ODTS-treated TFTs, V_TH_ increased, and the on-off current ratio decreased, which can be explained by the following: As the OTS or ODTS combined with Si-O-H, HCl was generated, and HCl still remained at 120 °C, possibly acting as a charge trap [28]. However, after annealing at T_A_ = 200 °C, the dipole formed by the aligned carbon chain reduced V_TH_ and improved the effective carrier mobility. The OTS-treated TFT was 1.3 times increase in effective carrier mobility (1.63–2.14 (cm^2^/V_S_), in saturation region). Especially, ODTS-treated TFTs showed 2.5 times larger (2.68–4.27 (cm^2^/V_S_), in saturation region) increase in effective carrier mobility compared with the pristine TFT. Through the above results, it was concluded that the doping of the OTS and ODTS treatments was effective.

Regarding the difference between the doping effects of OTS and ODTS, OTS and ODTS had carbon chain lengths of 8 and 18, respectively. The observed higher improvement by ODTS compared to that by OTS indicated the difference in the doping effect according to the length of the carbon chain; as a relatively larger dipole is formed by the long carbon chain, thereby controlling the doping effect [29,30,31].

### 3.2. Contact Resistance Analysis

**Figure 5:**
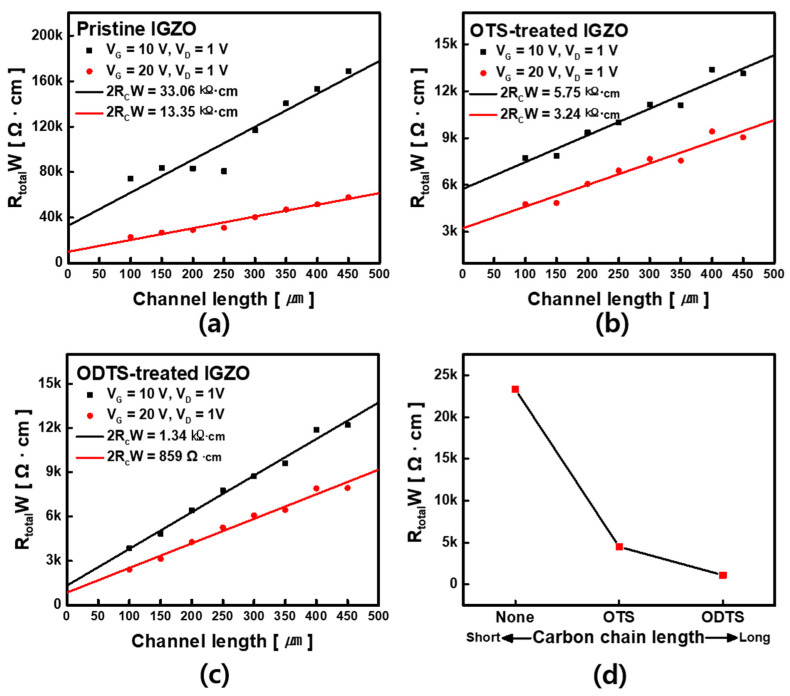
Contact resistance graph of pristine (**a**), OTS-treated (**b**) and ODTS-treated transistors; (**c**,**d**) Influence between length of carbon chain and contact resistance.

To investigate the doping effects in terms of contact resistance (R_C_), we used the transfer line method (TLM). Figure 5a shows a huge resistance, observed in the undoped transistor (2R_C_W = 33.06 kΩ·cm at V_G_ = 10 V; 2R_C_W = 13.35 kΩ·cm at V_G_ = 20 V). However, OTS- and ODTS-doped transistors provided a lower Rc compared with the counterpart of the pristine TFTs (OTS-treated TFT: 2 R_C_·W = 5.75 kΩ·cm at V_G_ = 10 V and 2 R_C_·W = 3.24 kΩ·cm at V_G_ = 20 V, in Figure 5b) (ODTS-treated TFT: 2 R_C_W = 1.34 kΩ·cm at V_G_ = 10 V and 2 R_C_W = 859 Ω·cm at V_G_ = 20 V, in Figure 5c). The observed Rc reduction resulted from the thinner Schottky barrier by the dipole effect of the SAM treatment; the SAM bonded to the upper surface of the IGZO, thereby inducing energy band bending by the positive dipole effect, and the contact resistance was reduced due to the thinner Schottky barrier, which is consistent with the results of previous studies [29,30,31]. As a result, I_D_ increased due to the decrease in R_C_, thereby increasing the on–off current ratio occurring. Figure 5d shows the influence of the transition of the chain length and contact resistance. Based on the above results, it was concluded that R_C_ and the SAM treatment doping are significantly related. The electrical characteristics of the TFTs can be modulated based on the carbon chain length of the trichlorosilane carbon chains with the variable T_A_.

### 3.3. Contact Angle and Surface Energy Analysis

In this study, we analyzed the contact angle and surface energy. Deionized water and formamide were used to measure the contact angle and surface energy. The surface energy was derived from the equation below [32,33]:

(1) $$\gamma^{sv}=\gamma^{sl}+\gamma^{lv}\cdot cos\theta$$

where *θ* = contact angle, $\gamma^{sl}$ = the solid/liquid interfacial free energy, $\gamma^{sv}$ = the solid surface free energy, $\gamma^{lv}$ = the liquid surface free energy.

**Figure 6:**
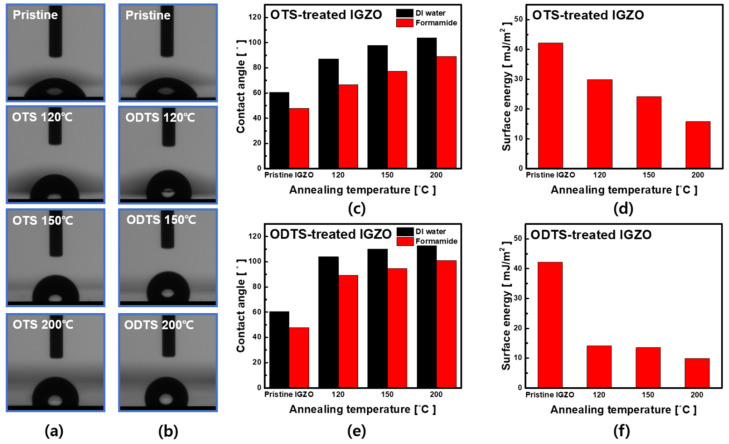
(**a**,**b**) The image of change in contact angle at OTS and ODTS. Change of contact angle by (**c**) OTS, (**e**) ODTS treatment and annealing. (**d**), (**f**) Surface energy according to the change in contact angle of each SAMs. The (**c**–**f**) graph is plotted as mean of 5 samples.

Figure 6 shows the transition of the contact angle and surface energy depending on the SAM treatment and T_A_. As shown in Figure 6a,b, the contact angle increased in the OTS and ODTS-treated IGZO surface more than the counterpart in the pristine IGZO surface [33,34] and increased further at T_A_ = 150 °C and 200 °C (i.e., the OTS-treated IGZO: 60.38–103.68°, the ODTS-treated IGZO: 60.38–112.70°, by DI water droplet), and the surface became hydrophobic as a function of T_A_, which was also supported by the extracted surface energy values; From the baseline of the surface energy (42.26 mJ/m^2^), the OTS treatment at T_A_ = 200 °C reduced the surface energy value to 15.76 mJ/m^2^ while the ODTS treatment at T_A_ = 200 °C reduced the surface energy value to 9.9 mJ/m^2^. These results indicated that both OTS and ODTS SAM treatment enabled the surface to be hydrophobic as the SAM molecules became aligned with increasing T_A_.

## 4. Conclusions

In summary, we demonstrated the systematic control of the SAM-based doping technique based on molecular chain length control and annealing temperature conditions (T_A_ = 120, 150 and 200 °C). By simply spin-coating OTS and ODTS on top of the prefabricated IGZO transistors, the n-doping effects were obtained. As T_A_ increased and the carbon chain length became larger, the electrical properties were enhanced, providing increased effective carrier mobility, lower V_TH_, and lower SS. Furthermore, the mechanism of the doping method was investigated by means of the contact resistance analysis and surface energy measurement. The control of the SAM doping through the study is expected to offer a helpful guideline for obtaining high-performance doping techniques using SAMs.
